# An Acylhydrazone-Based Fluorescent Sensor for Sequential Recognition of Al^3+^ and H_2_PO_4_^−^

**DOI:** 10.3390/ma14216392

**Published:** 2021-10-25

**Authors:** Donghwan Choe, Cheal Kim

**Affiliations:** Department of Fine Chemistry, Seoul National University of Science and Technology (SNUT), Seoul 136-742, Korea; ehdghksdl_@naver.com

**Keywords:** aluminum ion, dihydrogen phosphate, acylhydrazone, fluorescent chemosensor, sequential detection, calculations

## Abstract

A novel acylhydrazone-based fluorescent sensor **NATB** was designed and synthesized for consecutive sensing of Al^3+^ and H_2_PO_4_^−^. **NATB** displayed fluorometric sensing to Al^3+^ and could sequentially detect H_2_PO_4_^−^ by fluorescence quenching. The limits of detection for Al^3+^ and H_2_PO_4_^−^ were determined to be 0.83 and 1.7 μM, respectively. The binding ratios of **NATB** to Al^3+^ and **NATB**-Al^3+^ to H_2_PO_4_^−^ were found to be 1:1. The sequential recognition of Al^3+^ and H_2_PO_4_^−^ by **NATB** could be repeated consecutively. In addition, the practicality of **NATB** was confirmed with the application of test strips. The sensing mechanisms of Al^3+^ and H_2_PO_4_^−^ by **NATB** were investigated through fluorescence and UV–Visible spectroscopy, Job plot, ESI-MS, ^1^H NMR titration, and DFT calculations.

## 1. Introduction

Al^3+^, the third most abundant metallic element in nature [1,2], is broadly employed in daily life in packaging materials, pharmaceuticals, food additives, machinery, clinical medicines, and water purification [3,4]. Owing to its widespread usage, Al^3+^ could be readily accumulated in the body, which leads to the development of diverse diseases such as Parkinson’s and Alzheimer’s disease [5,6]. Dihydrogen phosphate (H_2_PO_4_^−^) is an important component related to many intercellular activities, such as signaling mediation, protein phosphorylation, enzymatic reactions, ion-channel regulation, and so on [7,8,9]. However, excessive agricultural use of phosphate causes eutrophication or massive algal growth, leading to a deficiency in oxygen levels [10,11,12]. For these reasons, there has been a strong demand for the development of sensing and detection methods for Al^3+^ and H_2_PO_4_^−^.

The traditional analytical methods reported for the analysis of cations and anions, such as ICP-AES, AAS, and electrochemical methods, have been largely restricted due to their expensive instruments, complicated procedures, and the need for highly trained operators [13,14,15]. In contrast, fluorescence methods have shown the advantages of cost-effectiveness, simplicity, easy operation, and high sensitivity [16,17,18]. While numerous fluorescent chemosensors for a single analyte have been reported, fluorescent chemosensors that allow the sequential sensing of multiple analytes with great selectivity and sensitivity are still needed [19,20,21] because they are more cost-effective, recyclable and practical [22,23,24]. Several fluorescent sensors have been addressed for consecutive sensing of Al^3+^ and several anions [25,26,27,28] or several cations and H_2_PO_4_^−^ [29,30,31]. In addition, Kumar et al. reported a fluorescent sensor for sequential sensing of Al^3+^ and H_2_PO_4_^−^/HSO_4_^−^ [32]. The practical importance of sequential sensing may have potential applications such as logic gates and molecular switches. Nevertheless, a sequential fluorescent sensor that can exclusively detect Al^3+^ and H_2_PO_4_^−^ has not been reported to date.

As Al^3+^ is a hard cation, chemosensors containing hard base units, such as nitrogen or oxygen atoms, prefer to coordinate with Al^3+^ [33,34,35]. In this regard, acylhydrazone derivatives, having oxygen and nitrogen atoms, are expected to be a suitable functional group to design an Al^3+^ chemosensor [36,37,38]. Naphthalene moieties have been widely applied for the design of fluorescent sensors because of their excellent photophysical properties as a fluorophore [39,40,41]. Hence, we expected that a compound including both acylhydrazone and naphthalene may operate as a fluorescence chemosensor for Al^3+^.

In the current study, we designed an acylhydrazone-based fluorescent sensor, **NATB**, which showed green fluorescence emissions with Al^3+^ and could sequentially detect H_2_PO_4_^−^ through fluorescence quenching with high sensitivity and selectivity. A sensing mechanism of **NATB** to Al^3+^ and H_2_PO_4_^−^ was illustrated by fluorescence and UV–Vis spectroscopy, Job plot, ESI-MS, ^1^H NMR titration, and calculations.

## 2. Experimental Section

### 2.1. Materials and Equipment

All solvents and reagents were commercially obtained from TCI (TCI, Nihonbashi-Honcho, Tokyo, Japan) and Sigma-Aldrich (MilliporeSigma, Burlington, MA, USA). NMR experiments were conducted using DMSO-*d_6_* as the solvent, and the data were recorded on a Varian spectrometer (Varian, Palo Alto, CA, USA). Fluorescence and UV–Visible spectra were measured with Perkin Elmer machines (Perkin Elmer, Waltham, MA, USA). The quantum yields of **NATB** and **NATB**-Al^3+^ were relatively determined with quinine (*Φ* = 0.54 in 1 × 10^−1^ M H_2_SO_4_) as a reference. ESI-MS data were recorded on a Thermo Finnigan machine (Thermo Finnigan LLC, San Jose, CA, USA).

### 2.2. Synthesis of N′-[(E)-(3-tert-butyl-2-hydroxyphenyl)methylidene]-3-hydroxynaphthalene-2-carbohydrazide (NATB)

The intermediate compound, 3-hydroxy-2-naphthohydrazide (**2**), was synthesized following a previously reported method [42]. The excess amounts of 3-(*tert*-butyl)-2-hydroxybenzaldehyde (**1**, 1.8 mmol) and 3-hydroxy-2-naphthohydrazide (**2**, 0.3 mmol) were mixed in absolute EtOH (10 mL) with a catalytic amount of HCl and stirred at room temperature for 1 day. A yellow precipitate was filtered, rinsed with cold absolute EtOH, and dried (77.2 mg, 70.1%); ^1^H NMR in DMSO-*d_6_*: δ 12.42 (s, 1H), 12.24 (s, 1H), 11.19 (s, 1H), 8.63 (s, 1H), 8.45 (s, 1H), 7.93 (d, 1H), 7.77 (d, 1H), 7.53 (t, 1H), 7.38 (t, 1H), 7.35 (s, 1H), 7.32 (d, 1H), 7.30 (d, 1H), 6.91 (t, 1H), 1.43 (s, 9H). ^13^C NMR in DMSO-d6: δ 163.23 (1C), 156.90 (1C), 153.75 (1C), 151.45 (1C), 136.36 (1C), 135.84 (1C), 130.31 (1C), 129.53 (1C), 128.59 (1C), 128.54 (1C), 128.20 (1C), 126.69 (1C), 125.75 (1C), 123.75 (1C), 119.86 (1C), 118.70 (1C), 117.54 (1C), 110.54 (1C), 34.39 (1C), 29.16 (3C). ESI-MS (*m/z*): [**NATB** + H^+^]^+^ calcd 363.17, found 363.04.

### 2.3. Preparation of Spectroscopic Experiments

An **NATB** stock (10 mM) was prepared in DMSO. The stock solutions (20 mM) of varied cations were prepared using their nitrate salts (Al^3+^, Na^+^, Cr^3+^, Fe^2+^, Ca^2+^, Cd^2+^, Zn^2+^, Pb^2+^, Co^2+^, Cu^2+^, In^3+^, Mn^2+^, Ga^3+^, Ni^2+^, Mg^2+^, Ag^+^, Hg^2+^ and K^+^) or perchlorate salt (Fe^3+^). The concentrated solutions (20 mM) of varied anions were prepared using their tetrabutylammonium salts (H_2_PO_4_^−^, SCN^−^, BzO^−^, N_3_^−^, OAc^−^ and NO_2_^−^), tetraethylammonium salts (F^−^, Cl^−^, Br^−^, I^−^ and CN^−^), sodium salts (S^2−^ and ClO^−^), or potassium salts (HPO_4_^2−^, PO_4_^3−^, HSO_4_^−^ and P_2_O_7_^4−^(PPi)). All spectroscopic experiments were conducted in MeOH.

### 2.4. Competitive Experiments

For Al^3+^, 6 μL (10 mM) of an **NATB** stock in DMSO was mixed into MeOH (2 mL) to make 30 μM. A total of 4.5 μL of various cations (20 mM) in DMF was diluted in **NATB** to make 45 μM. Finally, 4.5 μL (20 mM) of an Al^3+^ stock in DMF was mixed into each solution to produce 45 μM, and their fluorescent spectra were measured.

For H_2_PO_4_^−^, 6 μL (10 mM) of an **NATB** stock in DMSO and 4.5 μL (20 mM) of an Al^3+^ stock in DMF were diluted into MeOH (2 mL) to produce 30 μM of **NATB**-Al^3+^. We added 4.5 μL of various anions (20 mM) in H_2_O to **NATB**-Al^3+^ to produce 45 μM. A total of 4.5 μL (20 mM) of an H_2_PO_4_^−^ stock was diluted into each solution to produce 45 μM. Their fluorescent spectra were measured.

### 2.5. Determination of Association Constant (K)

The association constant (*K*) was calculated using Li’s method [43]. If the ligand (*L*) and the analyte (*M*) form an m-n complex, *M_m_L_n_*, the equilibrium constant of the corresponding complex, *K*, can be expressed by the following equation:

$${\left[M\right]}^{m}=\frac{1}{nK}\frac{1}{{\left[L\right]}_{T}^{n-1}}\frac{1-\alpha }{\alpha^{n}}$$

where,

$$\left[M\right]=the\;concentration\;of\;analyte\;{\left[L\right\}}_{T}=the\;total\;concentration\;of\;ligand$$

and α could be described as:

$$\alpha =\frac{I-I_{max}}{I_{min}-I_{max}}$$

where,

I=the fluorescence intensity of complex

### 2.6. Calculations

Calculations were achieved with the Gaussian 16 program [44]. Optimal geometries of **NATB** and **NATB**-Al^3+^ were provided with the DFT method [45,46]. B3LYP was selected as the hybrid functional basis set. The 6–31G(d,p) basis set was implemented to all atoms except Al^3+^ [47,48], and the LANL2DZ basis set was employed for applying ECP to Al^3+^ [49,50,51]. No imaginary frequency was found in the optimized states of **NATB** or **NATB**-Al^3+^, indicating their local minima. The solvent effect of MeOH was considered with IEFPCM [52]. Based on the energy-optimized structures of **NATB** and **NATB**-Al^3+^, the plausible UV–Vis transition states were calculated by the TD-DFT method with 20 lowest singlet states.

## 3. Results and Discussion

**Scheme 1:**
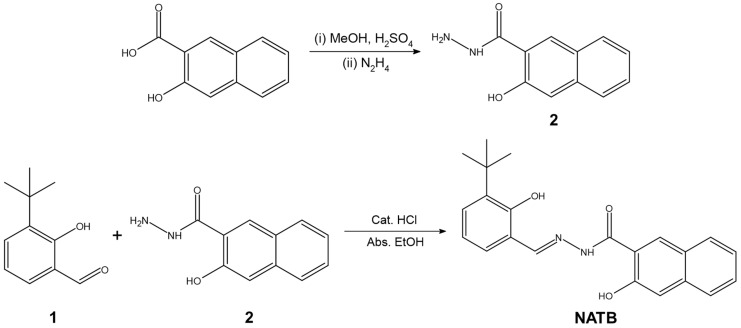
Synthesis of NATB.

The synthesis of **NATB** was conducted as depicted in Scheme 1. The condensation reaction of 3-(*tert*-butyl)-2-hydroxybenzaldehyde (**1**) and 3-hydroxy-2-naphthohydrazide (**2)** produced the desired product, *N’*-[(E)-(3-tert-butyl-2-hydroxyphenyl)methylidene]-3-hydroxynaphthalene-2-carbohydrazide (**NATB**), which was verified with ^1^H NMR, ^13^C NMR (Figures S1 and S2), and ESI-MS.

### 3.1. Spectroscopic Examination of NATB to Al^3+^

**Figure 1:**
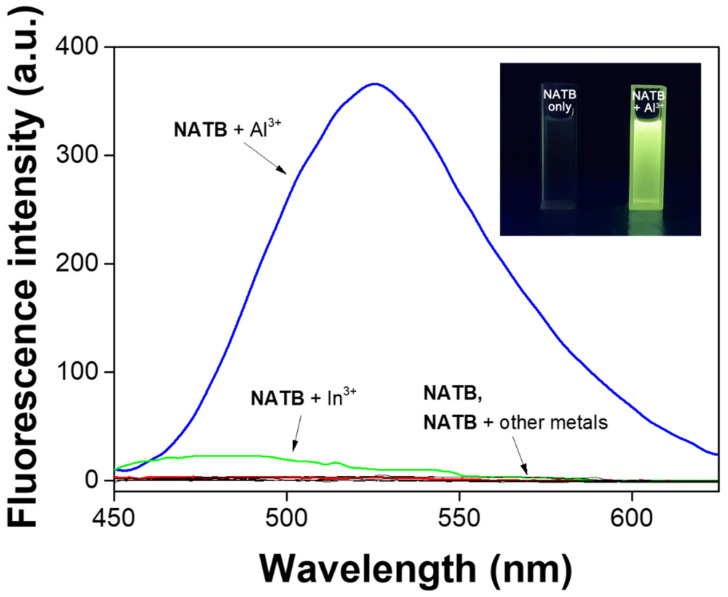
Fluorescence changes of **NATB** (30 μM) with a variety of cations (1.5 equiv) in MeOH. Photograph: the fluorescent images of **NATB** and **NATB**-Al^3+^ (λ_ex_: 358 nm).

To confirm the fluorescence selectivity of **NATB**, the fluorescence emission was studied with a variety of cations in MeOH (Figure 1). As a result, **NATB** exhibited notable fluorescence emission at 526 nm with Al^3+^, while **NATB** and **NATB** with other cations showed negligible or no fluorescence emission (λ_ex_ = 358 nm). These outcomes demonstrated that **NATB** could be utilized as a fluorescent probe for the selective sensing of Al^3+^. On the other hand, **NATB** was soluble in aqueous media, but it did not show any selectivity to Al^3+^. In addition, the fluorescence emission of **NATB** was examined with various anions including dihydrogen phosphate. **NATB** had no selectivity for the anions.

**Figure 2:**
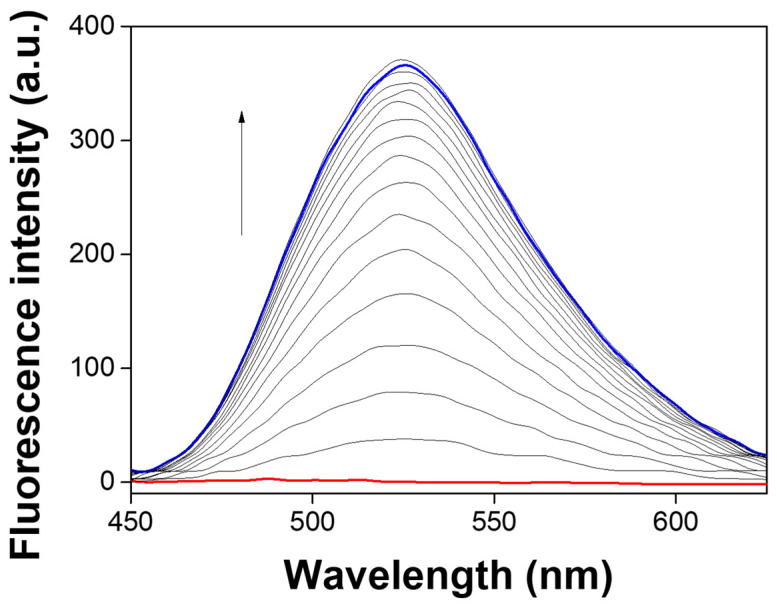
Fluorescence titration of **NATB** (30 μM) with varied amounts of Al^3+^ (0–1.5 equiv) in MeOH.

**Figure 3:**
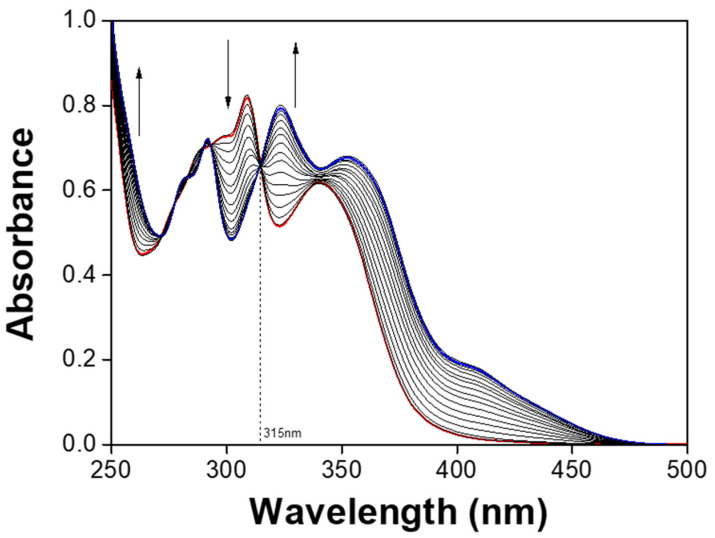
UV–Vis changes of **NATB** (30 μM) with varied amounts of Al^3+^ (0–1.5 equiv) in MeOH.

To check the concentration-dependent properties of **NATB** to Al^3+^, fluorescence titration was carried out (Figure 2). **NATB** exhibited little fluorescence with a tiny quantum yield (*Φ* = 0.008). However, the continuous increase in Al^3+^ up to 1.5 equiv significantly enhanced the green fluorescence emission at 526 nm (*Φ* = 0.162). UV–Vis spectrometry was also conducted with Al^3+^ to examine its photophysical characteristics (Figure 3). Upon the addition of Al^3+^, the absorption of 310 nm clearly decreased, while a new absorption of 325 nm constantly increased up to 1.5 equiv. An explicit isosbestic point was observed at 315 nm, verifying that the coordination of **NATB** with Al^3+^ produced a stable complex.

A 1:1 stoichiometric coordination between **NATB** and Al^3+^ was suggested by the Job plot experiment (Figure S3), which was explicitly supported by ESI-MS analysis (Figure S4). The positive ion mass displayed a large peak of 596.16 (*m/z*), which was correlated to [**NATB**(-H^+^) + Al^3+^ + 2 DMF + NO_3_^−^]^+^ (calcd. 596.23). The association constant (*K*) of **NATB**-Al^3+^ was confirmed to be 3.6 × 10^4^ M^−1^ (Figure S5) based on Li’s method [43]. The detection limit of **NATB** toward Al^3+^ was 0.83 μM, based on 3σ/slope (Figure S6).

**Figure 4:**
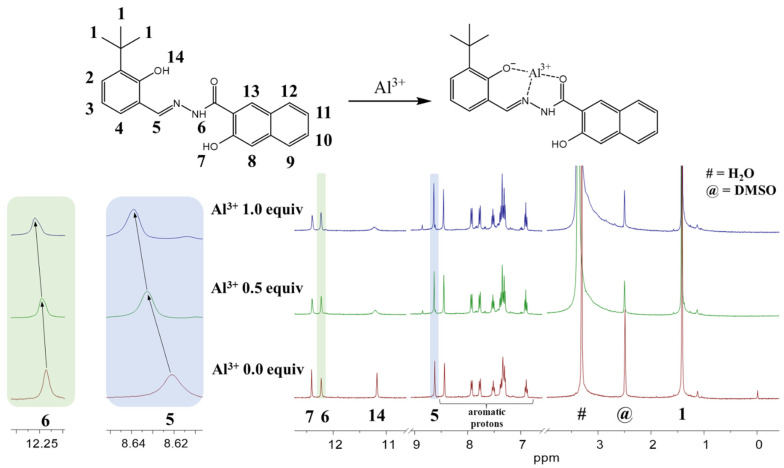
^1^H NMR titration of **NATB** with Al^3+^ in DMSO-*d_6_*.

**Scheme 2:**
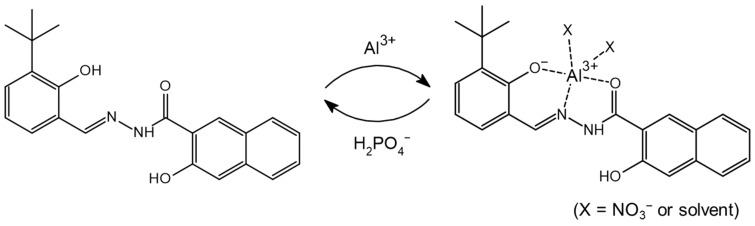
Sequential recognition mechanism of Al^3+^ and H_2_PO_4_^−^ by **NATB**.

The ^1^H NMR titrations were achieved to investigate the binding mechanism of **NATB** toward Al^3+^ (Figure 4). Upon the addition of Al^3+^ to **NATB**, the proton H_14_ continually disappeared and the protons H_5_ and H_6_ were deshielded. These results indicate that the deprotonated oxygen on the *tert*-butylphenol group and the oxygen and nitrogen on the acylhydrazone group may be coordinated to Al^3+^ (Scheme 2).

**Figure 5:**
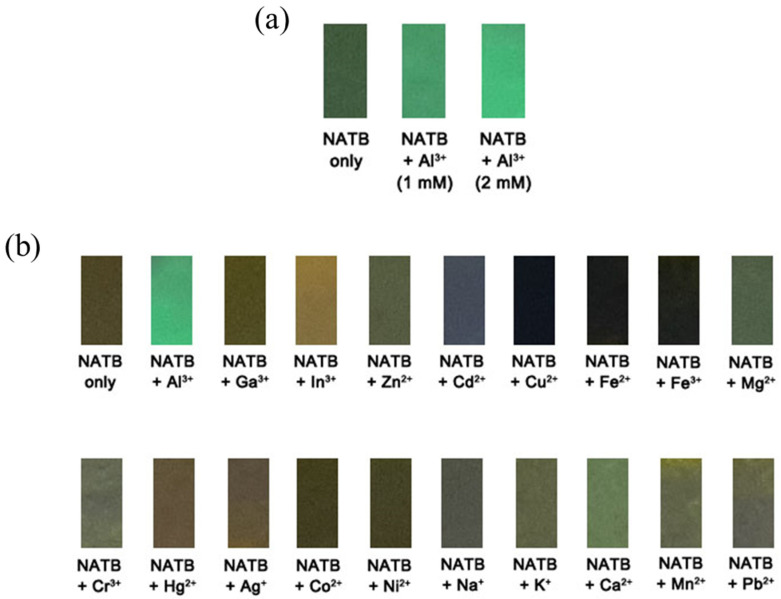
Detection of Al^3+^ by **NATB**-coated test kits (10 mM). (**a**) **NATB**-coated test kits immersed in the solution of different Al^3+^ concentrations; (**b**) **NATB**-coated test kits immersed in 2 mM of various cation solutions.

To verify the practicability of **NATB** as a probe for Al^3+^, an interference experiment was conducted (Figure S7). **NATB** could detect Al^3+^ with other cations without significant interferences, except for In^3+^, Fe^3+^ and Cu^2+^. These three cations bound more tightly to **NATB** than Al^3+^. For the practical application of **NATB**, test kits were prepared by immersing filter paper strips in the **NATB** solution. When **NATB**-coated test kits were immersed in a range of concentrations of Al^3+^ solutions, the obvious green fluorescence emission showed up above 2 mM of Al^3+^ under UV light (Figure 5a). However, the fluorescence was not displayed when those strips were applied to the same concentration of other cations (Figure 5b). These results indicate the potential applications of **NATB** in easily recognizing Al^3+^ without any complicated tools.

### 3.2. Calculations

**Figure 6:**
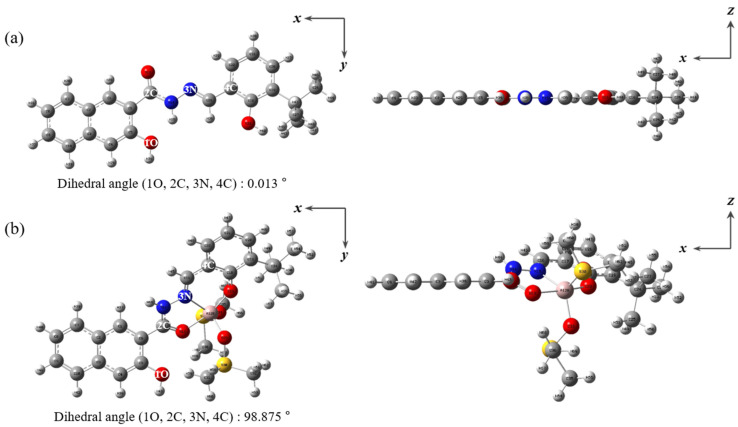
Energy-optimized forms of (**a**) **NATB** and (**b**) **NATB**-Al^3+^.

To comprehend the Al^3+^-sensing property of **NATB**, DFT calculations were performed with the Gaussian 16 program (Figure 6). As the Job plot, ESI-MS, and ^1^H NMR titration implied a 1:1 stoichiometric coordination of **NATB** with Al^3+^, all calculations were conducted with 1:1 stoichiometry. **NATB** showed a dihedral angle of 0.013° (1O, 2C, 3N, and 4C) with a planar structure (Figure 6a). The coordination of **NATB** with Al^3+^ distorted its structure, showing a dihedral angle of 98.875° (Figure 6b).

Based on the energy-minimized structures of **NATB** and **NATB**-Al^3+^, TD-DFT calculations were conducted to inspect the transition energies and molecular orbitals. **NATB** featured the main absorption induced from the HOMO → LUMO (347.28 nm), showing intra-charge transfer (ICT) transition from the *tert*-butylphenol to the naphthol (Figure S8). The major absorption of **NATB**-Al^3+^ derived from the HOMO-1 → LUMO transition (412.27 nm) also showed a similar ICT transition (Figures S9 and S10). The reduction in the energy gap was consistent with the red-shift of the experimental absorption. These outcomes led us to conclude that the fluorescence turn-on mechanism of **NATB** to Al^3+^ may be a chelation-enhanced fluorescence (CHEF) effect [53]. Based on experimental data and theoretical calculations, an appropriate binding structure of **NATB**-Al^3+^ is proposed in Scheme 2.

### 3.3. Spectroscopic Examination of NATB-Al^3+^ to H_2_PO_4_^−^

**Figure 7:**
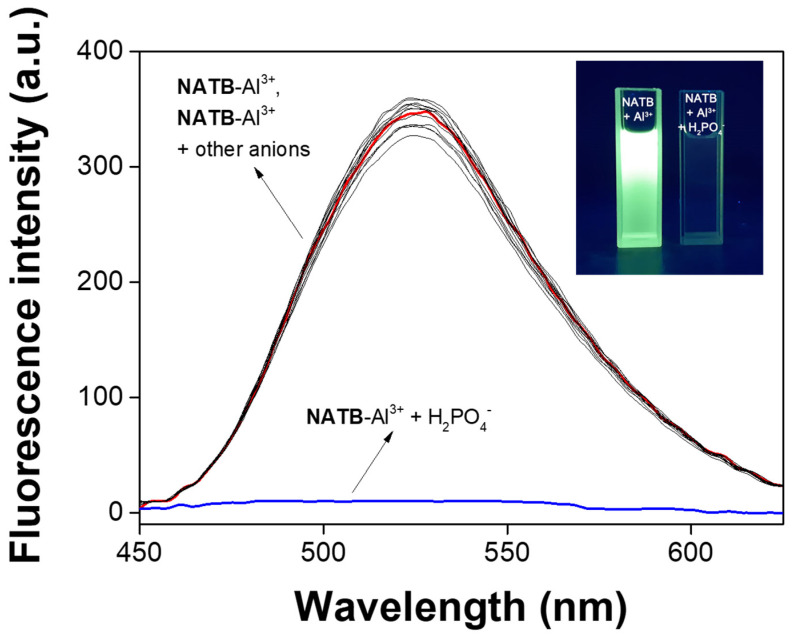
Fluorescence changes of **NATB**-Al^3+^ (30 μM) with various anions (45 μM) in MeOH (λ_ex_: 358 nm). Photograph: the fluorescent images of **NATB**-Al^3+^ and **NATB**-Al^3+^-H_2_PO_4_^−^ (λ_ex_: 358 nm).

We studied the fluorescence selectivity of **NATB**-Al^3+^ to a range of anions such as H_2_PO_4_^−^, Cl^−^, CN^−^, OAc^−^, F^−^, ClO^−^, I^−^, N_3_^−^, BzO^−^, SCN^−^, Br^−^, NO_2_^−^, S^2−^, HPO_4_^2−^, PO_4_^3−^, HSO_4_^−^, and PPi in MeOH (Figure 7). Most of the anions did not affect the fluorescence emission of **NATB**-Al^3+^, while the addition of H_2_PO_4_^−^ toward **NATB**-Al^3+^ resulted in significant fluorescence quenching (λ_ex_ = 358 nm). The result demonstrated that **NATB**-Al^3+^ could be used as a chemosensor for H_2_PO_4_^−^ with fluorescence quenching.

**Figure 8:**
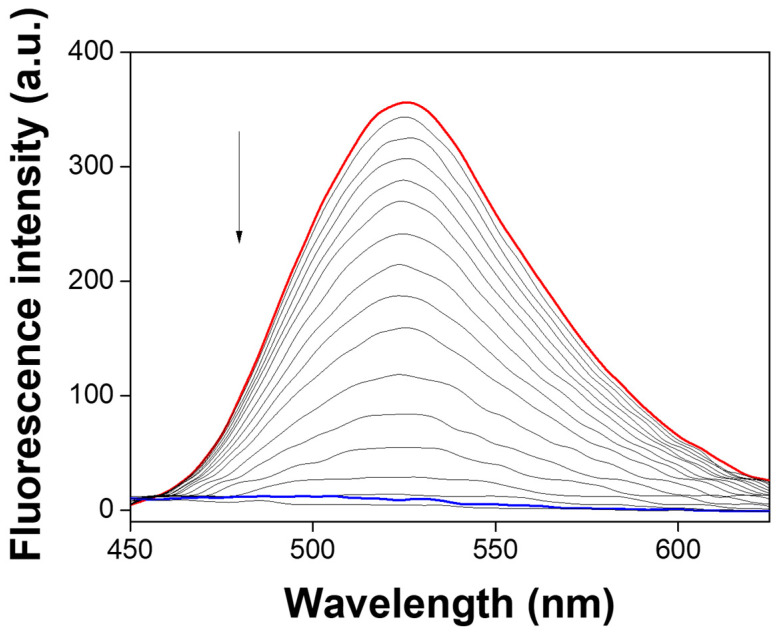
Fluorescence titration of **NATB**-Al^3+^ (30 μM) with various amounts of H_2_PO_4_^−^ (0–1.5 equiv) in MeOH.

**Figure 9:**
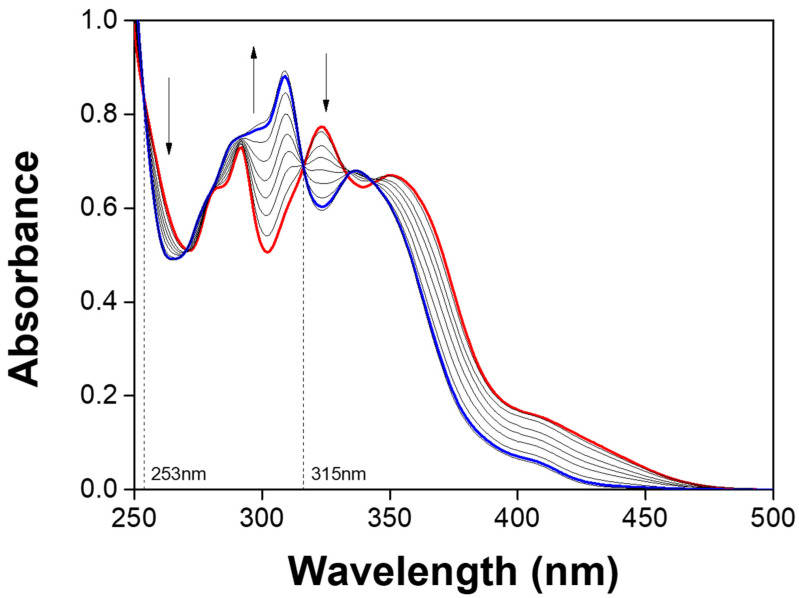
UV–Vis changes of **NATB**-Al^3+^ (30 μM) with various amounts of H_2_PO_4_^−^ (0–1.5 equiv) in MeOH.

The fluorescence titration experiments were conducted to verify the fluorescence quenching ability of H_2_PO_4_^−^ toward **NATB**-Al^3+^ (Figure 8). The fluorescence of **NATB**-Al^3+^ consistently diminished with the addition of H_2_PO_4_^−^ up to 1.5 equiv (*Φ* = 0.005). UV–Vis spectroscopy showed that the continuous addition of H_2_PO_4_^−^ increased the absorbance at 310 nm, while those at 270 and 325 nm decreased with the explicit isosbestic points at 253 and 315 nm (Figure 9). The UV–Vis spectrum of H_2_PO_4_^−^ with **NATB**-Al^3+^ is analogous to that of free **NATB**, implying that the addition of H_2_PO_4_^−^ released Al^3+^ from the **NATB**-Al^3+^ complex (Figure S11).

The stoichiometry of H_2_PO_4_^−^ toward **NATB**-Al^3+^ was determined by the Job plot experiment (Figure S12), which exhibited a 1:1 stoichiometry. The mass spectral analysis displayed a peak of 395.06 (*m*/*z*), which demonstrated the regeneration of **NATB** ([**NATB** + H^+^ + MeOH]^+^; calcd. 395.20) (Figure S13). These outcomes supported the mechanism that the addition of H_2_PO_4_^−^ released Al^3+^ from **NATB**-Al^3+^, which resulted in the loss of fluorescence. Based on Li’s method [43], the association constant (*K*) for H_2_PO_4_^−^ with **NATB**-Al^3+^ was calculated as 1.2 × 10^4^ M^−1^ (Figure S14). The detection limit of **NATB**-Al^3+^ toward H_2_PO_4_^−^ was determined as 1.7 μM, based on 3σ/slope (Figure S15). Importantly, **NATB** is the first fluorescent sensor for the consecutive sensing of Al^3+^ and H_2_PO_4_^−^ (Table S1). On the other hand, **NATB** showed higher detection limits for Al^3+^ and H_2_PO_4_^−^ compared to Kumar’s work [32], but it could solely detect H_2_PO_4_^−^ without the interference of HSO_4_^−^.

**Figure 10:**
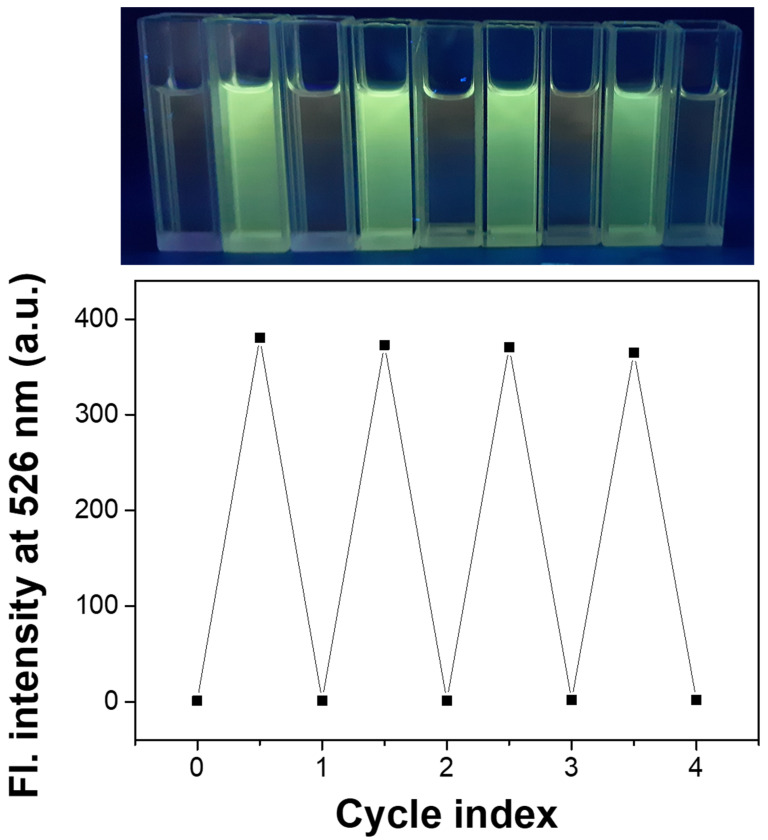
Change in fluorescence of **NATB** (30 μM) upon alternate addition of Al^3+^ and H_2_PO_4_^−^ in MeOH (λ_ex_: 358 nm).

The reversibility in the response of **NATB** was verified through the alternative additions of Al^3+^ and H_2_PO_4_^−^ (Figure 10). The fluorescence emission of **NATB** repeated its enhancing and quenching processes several times without fluorescence efficiency loss. To verify that **NATB**-Al^3+^ is an effective fluorescence probe for H_2_PO_4_^−^, the interference of other anions was tested (Figure S16). The results indicated that the presence of other anions (1.5 equiv) did not interfere with the fluorescence quenching of **NATB**-Al^3+^ toward H_2_PO_4_^−^.

## 4. Conclusions

An acylhydrazone-based chemosensor **NATB** was developed and its sequential recognition of Al^3+^ and H_2_PO_4_^−^ was studied. **NATB** showed a strong fluorescence increase with Al^3+^, and its complex **NATB**-Al^3+^ sequentially detected H_2_PO_4_^−^ by releasing Al^3+^ with turn-off fluorescence. Importantly, **NATB** is the first sequential fluorescent probe for selective sensing of Al^3+^ and H_2_PO_4_^−^. Detection limits of **NATB** for Al^3+^ and H_2_PO_4_^−^ were calculated as 0.83 and 1.7 μM, respectively, based on 3σ/slope. **NATB** could repeat sequential recognition of Al^3+^ and H_2_PO_4_^−^ several times and could be applied to detect Al^3+^ in test strips. The sensing mechanism of **NATB** toward Al^3+^ and H_2_PO_4_^−^ was demonstrated with a Job plot, ESI-MS, ^1^H NMR spectroscopy, and theoretical calculations. The detection mechanism of **NATB** toward Al^3+^ is suggested to be a CHEF effect through DFT calculations.

## Supplementary Materials

The following are available online at https://www.mdpi.com/article/10.3390/ma14216392/s1, Table S1: Examples of chemosensors for successive detection related to Al^3+^ or H_2_PO_4_^−^ or both; Figure S1: ^1^H NMR spectrum of **NATB** in DMSO-*d_6_*; Figure S2: ^13^C NMR spectrum of **NATB** in DMSO-*d_6_*; Figure S3: Job plot for the binding of **NATB** with Al^3+^ (50 μM) in MeOH. Fluorescence intensity at 526 nm is plotted as a function of the molar ratio of [Al^3+^]/([Al^3+^] + [**NATB**]); Figure S4: Positive-ion ESI mass spectrum of **NATB** (100 μM) in MeOH upon the addition of 1 equiv of Al^3+^ in DMF; Figure S5: Li’s equation plot (at 526 nm) of **NATB** (30 µM) in MeOH, based on fluorescence titration, assuming 1:1 stoichiometry for association between **NATB** and Al^3+^; Figure S6: Calibration curve of **NATB** as a function of Al^3+^ concentration in MeOH. [**NATB**] = 30 μM and [Al^3+^] = 0–18 μM (λ_ex_ = 358 nm); Figure S7: Competitive experiments of **NATB** (30 µM) toward Al^3+^ (45 µM) in the presence of other metal ions (45 µM, λ_ex_ = 358 nm) in MeOH; Figure S8: (a) The theoretical excitation energies and the experimental UV–Vis spectrum of **NATB.** (b) The major electronic transition energies and molecular orbital contributions of **NATB**; Figure S9: (a) The theoretical excitation energies and the experimental UV–Vis spectrum of **NATB**-Al^3+^. (b) The major electronic transition energies and molecular orbital contributions of **NATB**-Al^3+^; Figure S10: The major molecular orbital transitions and excitation energies of **NATB** and **NATB**-Al^3+^; Figure S11: UV–Vis spectra of **NATB** and **NATB**-Al^3+^ with H_2_PO_4_^−^ in MeOH, respectively; Figure S12: Job plot for the stoichiometry of **NATB**-Al^3+^ with H_2_PO_4_^−^ (30 μM) in MeOH. Fluorescence intensity at 526 nm is plotted as a function of the molar ratio of [**NATB**-Al^3+^]/([**NATB**-Al^3+^] + [H_2_PO_4_^−^]); Figure S13: Positive-ion ESI mass spectrum of **NATB**-Al^3+^ (100 μM) in MeOH upon the addition of 1 equiv of H_2_PO_4_^−^ in H_2_O; Figure S14: Li’s equation plot (at 526 nm) of **NATB**-Al^3+^ (30 µM) based on fluorescence titration in MeOH, assuming 1:1 stoichiometry for association between **NATB**-Al^3+^ and H_2_PO_4_^−^; Figure S15: Calibration curve of **NATB**-Al^3+^ in MeOH as a function of H_2_PO_4_^−^ concentration. [**NATB**-Al^3+^] = 30 μM and [H_2_PO_4_^−^] = 0.0–18.0 μM (λ_ex_ = 358 nm); Figure S16: Interference studies of **NATB**-Al^3+^ (30 µM) toward H_2_PO_4_^−^ (45 µM) in the presence of other anions (45 µM, λ_ex_ = 358 nm) in MeOH.
