# Supplementary material for: An Acylhydrazone-Based Fluorescent Sensor for Sequential Recognition of Al3+ and H2PO4−
Source: Materials (Basel). 2021 Oct 25;14(21):6392. doi: 10.3390/ma14216392 (PMC8585233; doi:10.3390/ma14216392)
Supplement: Supplementary file 1 [file materials-14-06392-s001.zip › materials-1412610-supplementary.pdf]

Supplementary Materials

An Acylhydrazone-Based Fluorescent Sensor for Sequential Recognition of Al^{3+} and $H_2PO_4^-$

Donghwan Choe and Cheal Kim *

Department of Fine Chem., Seoul National University of Science and Technology (SNUT), Seoul 136-742, Korea; ehdghksdl_@naver.com

* Correspondence: chealkim@snut.ac.kr; Tel.: +82-2-972-6673; Fax: +82-2-981-9147

Table S1. Examples of chemosensors for successive detection related to Al^{3+} or $H_2PO_4^-$ or both.

Structure	Analytes	Detection limit for Al^{3+}	Detection limit for $H_2PO_4^-$	Solvent	Reference
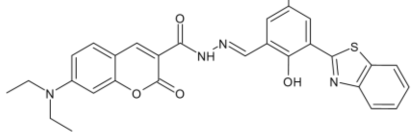	Al^{3+}, PPi	1.6×10^{-7} M	-	DMSO/HEPES (v/v = 4:1, pH = 7.4)	[1]
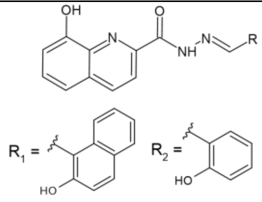	Al^{3+}, F^-	4.2×10^{-7} M (R_1) 1.5×10^{-7} M (R_2)	-	DMSO/H_2O (v/v = 1:2)	[2]
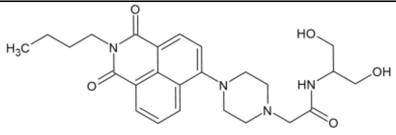	Al^{3+}, ClO^-	2.0×10^{-8} M	-	MeOH	[3]
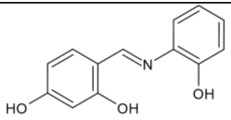	Al^{3+}, PO_4^{3-}	3.8×10^{-9} M	-	Water	[4]
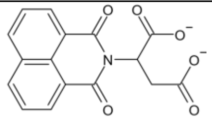	Fe^{3+}, $H_2PO_4^-$	-	5.3×10^{-6} M	Water	[5]
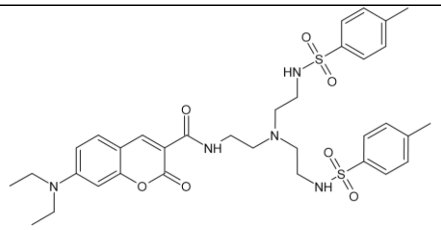	Cu^{2+}, $H_2PO_4^-$	-	1.6×10^{-6} M	MeCN/HEPES (v/v = 9:1, pH = 7.3)	[6]
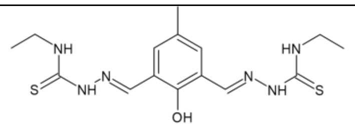	Zn^{2+}, $H_2PO_4^-$	-	2.6×10^{-5} M	MeOH/HEPES (v:v = 4:1 pH = 7.2)	[7]
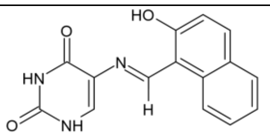	Al^{3+}, $H_2PO_4^-$, HSO_4^-	1.5×10^{-9} M	2.3×10^{-7} M	Water	[8]
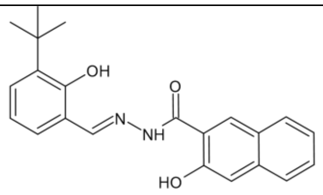	Al^{3+}, $H_2PO_4^-$	8.3×10^{-7} M	1.7×10^{-6} M	MeOH	This work

References

1. Li, S.; Cao, D.; Meng, X.; Hu, Z.; Li, Z.; Yuan, C.; Zhou, T.; Han, X.; Ma, W. A novel schiff base fluorescent probe based on coumarin and benzothiazole for sequential detection of Al^{3+} and PPi and its applicability in live cell imaging. *J. Photochem. Photobiol. A Chem.* **2020**, *392*, 112427, doi:10.1016/j.jphotochem.2020.112427.

2. Fu, J.; Li, B.; Mei, H.; Chang, Y.; Xu, K. Fluorescent schiff base probes for sequential detection of Al^{3+} and F^{-} and cell imaging applications. *Spectrochim. Acta Part A Mol. Biomol. Spectrosc.* **2020**, *227*, 117678, doi:10.1016/j.saa.2019.117678.

3. Sun, X.J.; Liu, T.T.; Fu, H.; Li, N.N.; Xing, Z.Y.; Yang, F. A Naphthalimide-Based Fluorescence "Off-on-Off" Chemosensor for Relay Detection of Al^{3+} and ClO^{-}. *Front. Chem.* **2019**, *7*, 549, doi:10.3389/fchem.2019.00549.

4. Huang, M.X.; Lai, J.P.; Sun, H.; Wu, W.Z. A simple, highly selective and ultra-sensitive "off-on-off" fluorescent chemosensor for successive detection of aluminum ion and phosphate in water samples. *Microchem. J.* **2019**, *151*, 104195, doi:10.1016/j.microc.2019.104195.

5. Zhang, Y.M.; Chen, X.P.; Liang, G.Y.; Zhong, K.P.; Yao, H.; Wei, T.B.; Lin, Q. A water-soluble fluorescent chemosensor based on Asp functionalized naphthalimide for successive detection Fe^{3+} and $H_2PO_4^-$. *Can. J. Chem.* **2018**, *96*, 363–370, doi:10.1139/cjc-2017-0451.

6. Meng, X.; Li, S.; Ma, W.; Wang, J.; Hu, Z.; Cao, D. Highly sensitive and selective chemosensor for Cu^{2+} and $H_2PO_4^-$ based on coumarin fluorophore. *Dyes and Pigments* **2018**, *154*, 194–198, doi:10.1016/j.dyepig.2018.03.002.

7. Purkait, R.; Mahapatra, A. Das; Chattopadhyay, D.; Sinha, C. An azine-based carbothioamide chemosensor for selective and sensitive turn-on-off sequential detection of Zn(II) and $H_2PO_4^-$, live cell imaging and INHIBIT logic gate. *Spectrochim. Acta Part A Mol. Biomol. Spectrosc.* **2019**, *207*, 164–172, doi:10.1016/j.saa.2018.09.019.

8. Kumar, A.; Kumar, V.; Upadhyay, K.K. An Al^{3+} and $H_2PO_4^-/HSO_4^-$ selective conformational arrest and bail to a pyrimidine-naphthalene anchored molecular switch. *Analyst* **2013**, *138*, 1891–1897, doi:10.1039/C3AN36697A.

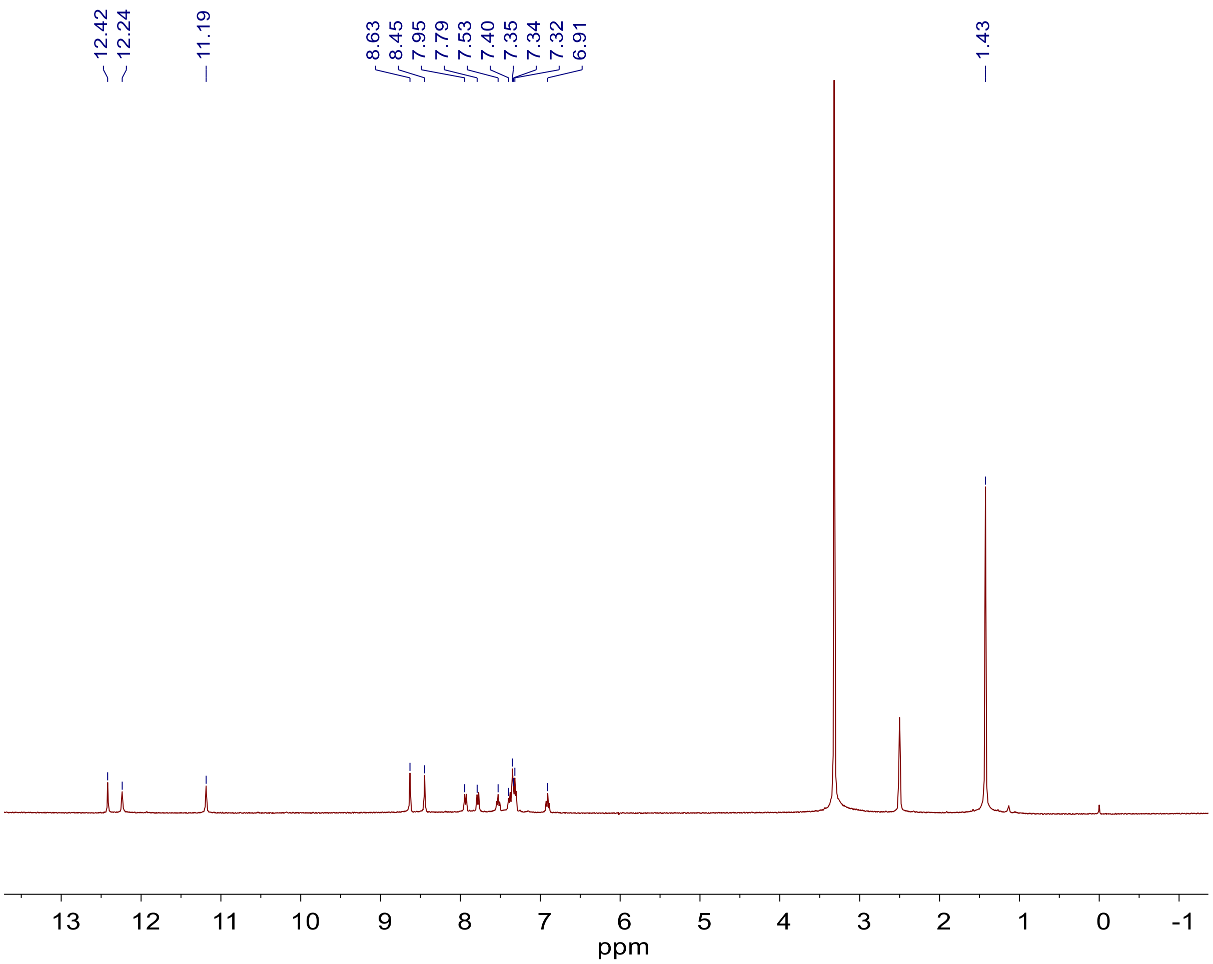

Figure S1. ^{1}H NMR spectrum of **NATB** in DMSO-d_6.

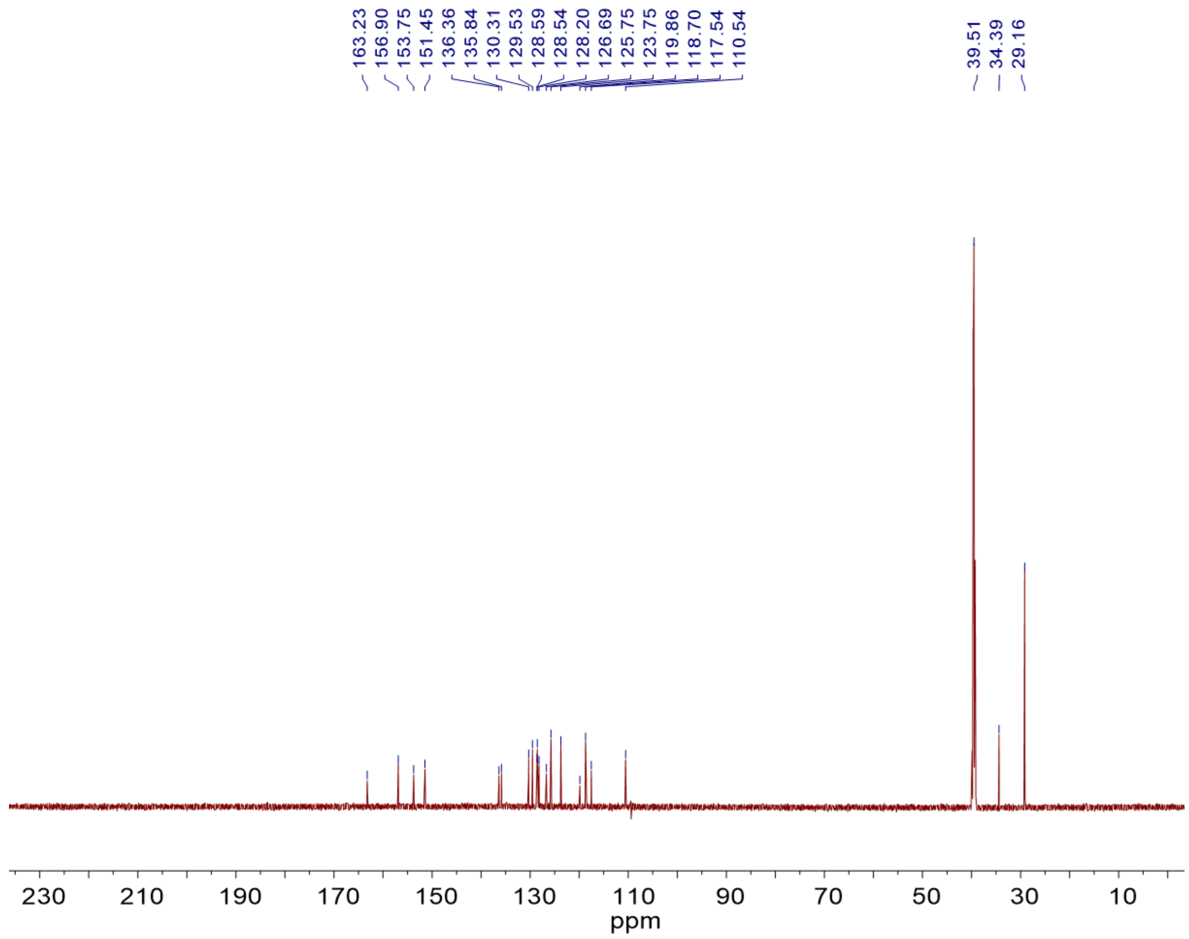

Figure S2. ^{13}C NMR spectrum of **NATB** in DMSO-d_6.

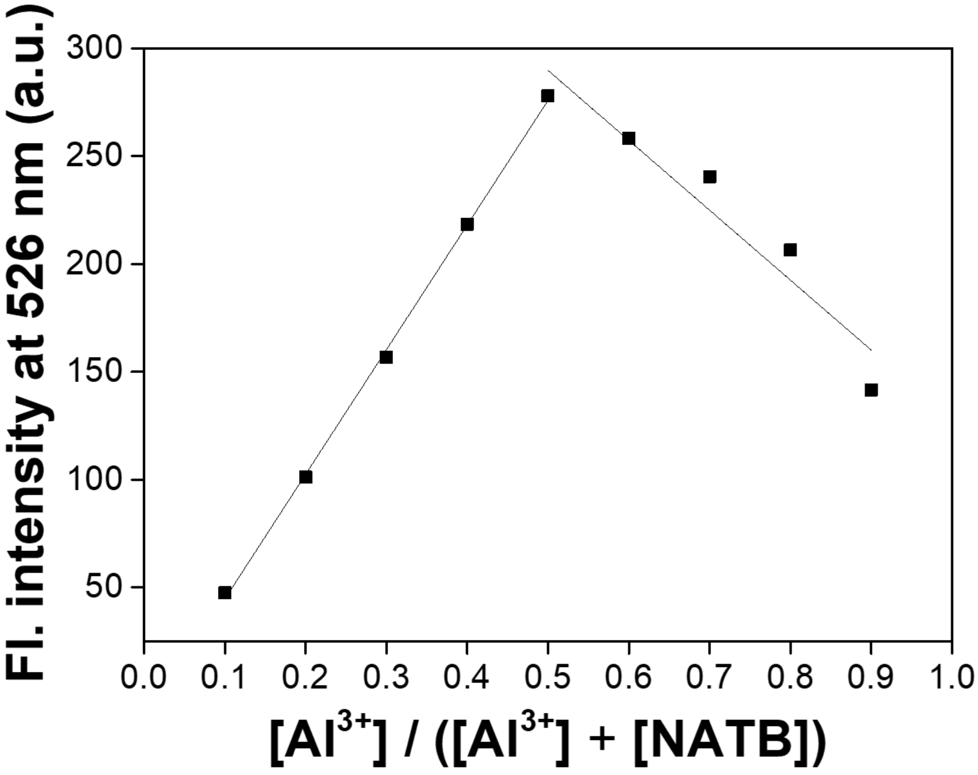

Figure S3. Job plot for the binding of **NATB** with Al^{3+} (50 μM) in MeOH. Fluorescence intensity at 526 nm was plotted as a function of the molar ratio of $[Al^{3+}]$ / ($[Al^{3+}]$ + [**NATB**]).

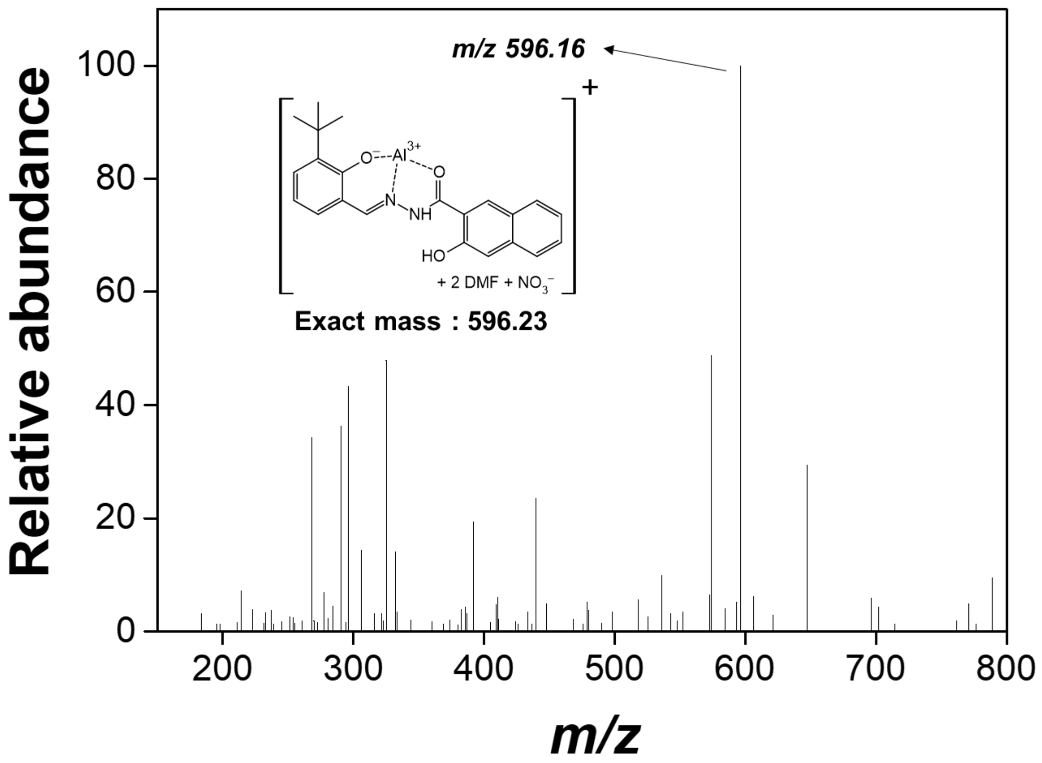

Figure S4. Positive-ion ESI-mass spectrum of **NATB** (100 μM) in MeOH upon the addition of 1 equiv of Al^{3+} in DMF.

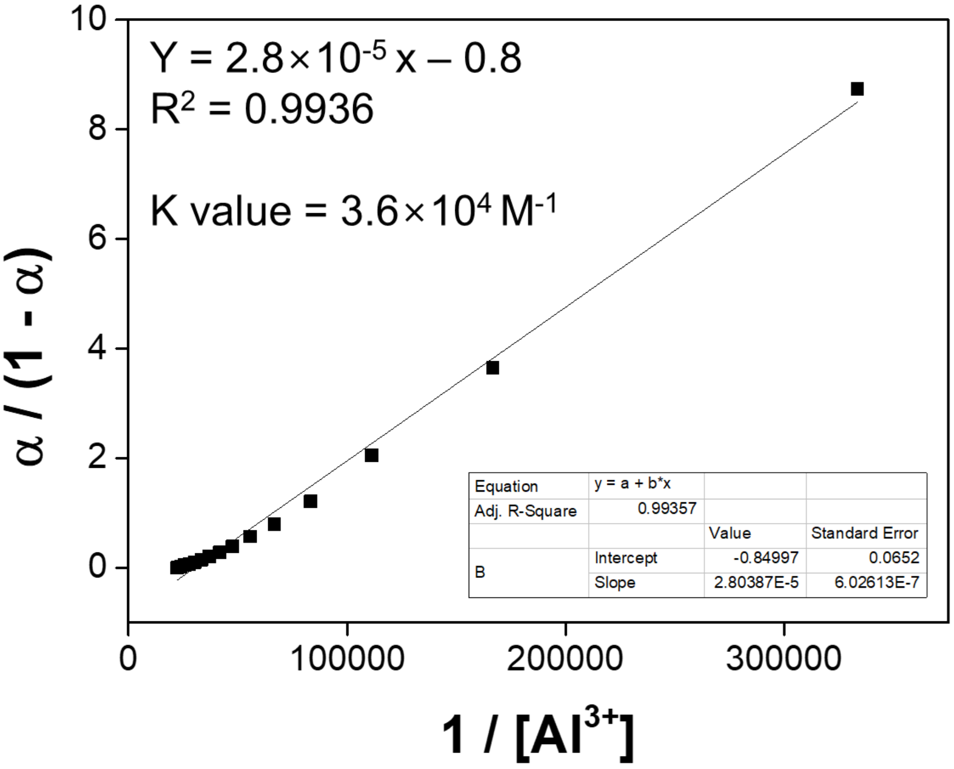

Figure S5. Li's equation plot (at 526 nm) of **NATB** (30 μM) in MeOH, based on fluorescence titration, assuming 1:1 stoichiometry for the association between **NATB** and Al^{3+}.

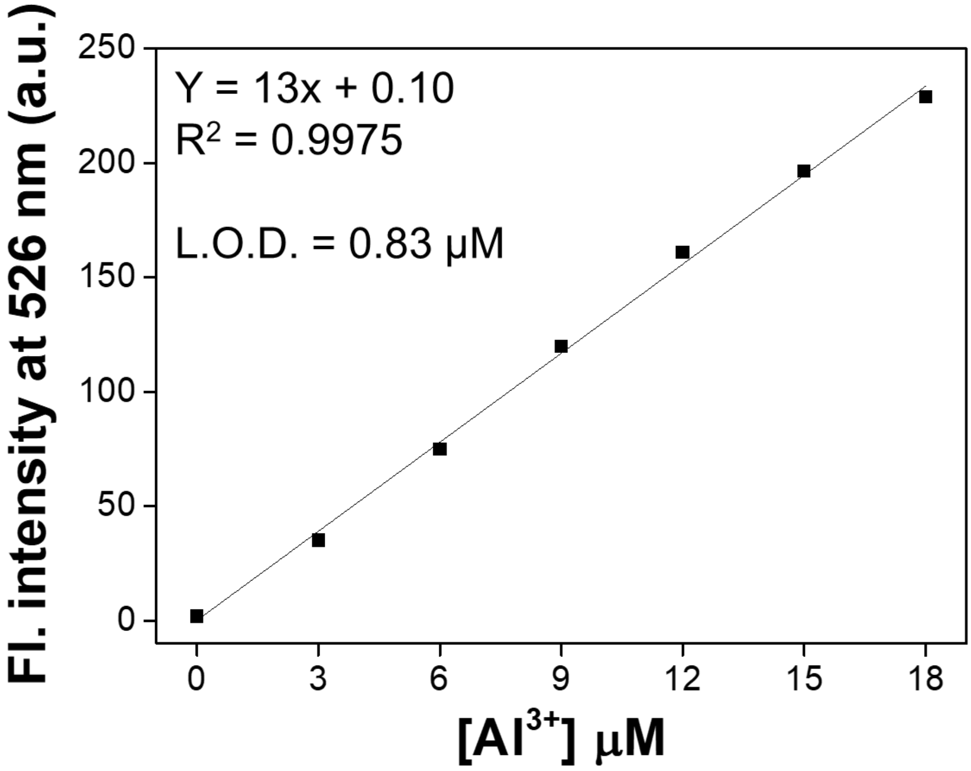

Figure S6. Calibration curve of **NATB** as a function of Al^{3+} concentration in MeOH. [**NATB**] = 30 μM and [Al^{3+}] = 0 - 18 μM (λ_{ex} = 358 nm).

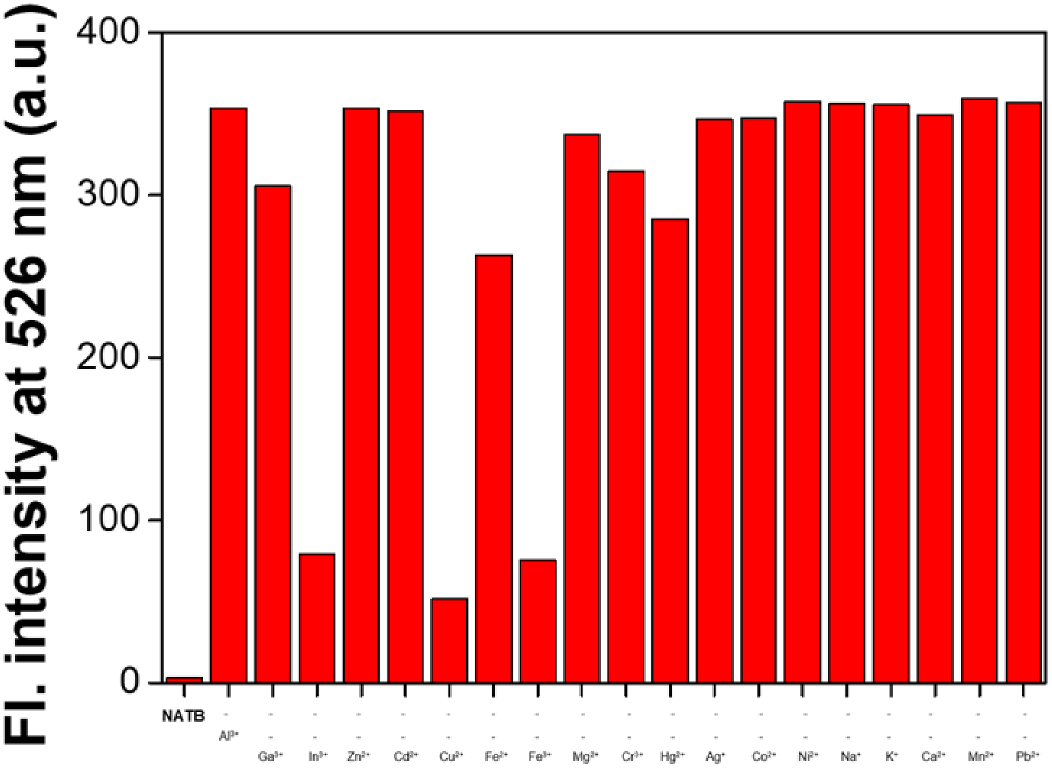

Figure S7. Competitive experiments of **NATB** (30 μM) toward Al^{3+} (45 μM) in the presence of other metal ions (45 μM, λ_{ex} = 358 nm) in MeOH.

(a)

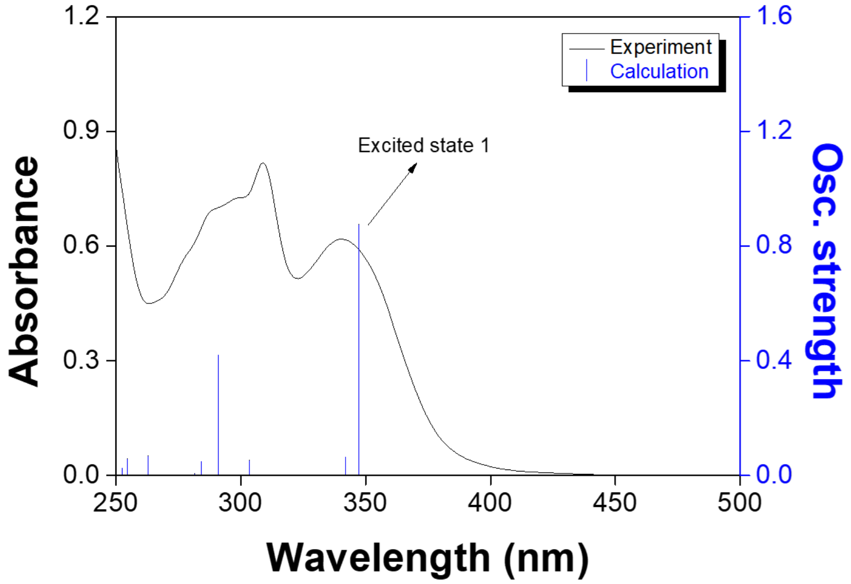

(b)

Excited state 1	Wavelength (nm)	Percent (%)	Main Character	Oscillator strength
HOMO → LUMO	347.28	91%	ICT	0.8757

Figure S8. (a) The theoretical excitation energies and the experimental UV-vis spectrum of **NATB**. (b) The major electronic transition energies and molecular orbital contributions of **NATB**.

(a)

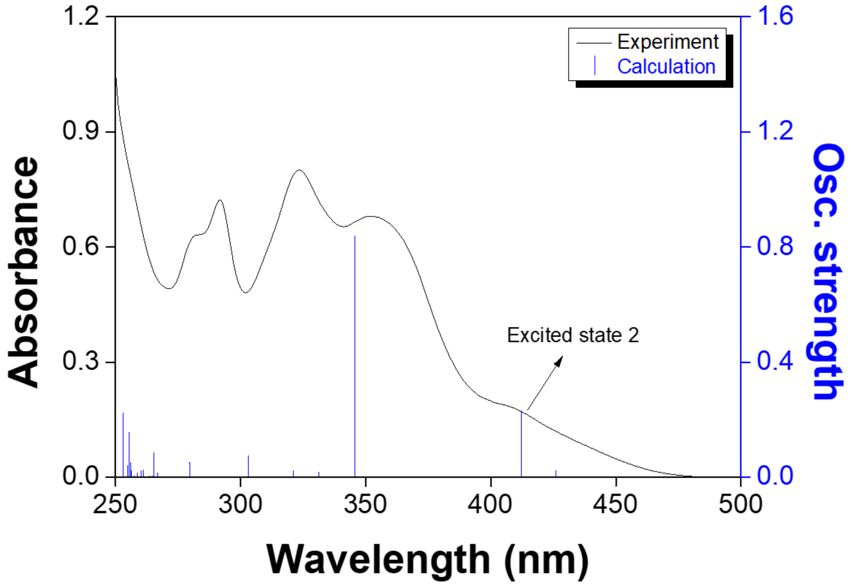

(b)

Excited state 2	Wavelength (nm)	Percent (%)	Main Character	Oscillator strength
HOMO-1 → LUMO	412.27	97%	ICT	0.2309

Figure S9. (a) The theoretical excitation energies and the experimental UV-vis spectrum of **NATB**-Al^{3+}. (b) The major electronic transition energies and molecular orbital contributions of **NATB**-Al^{3+}.

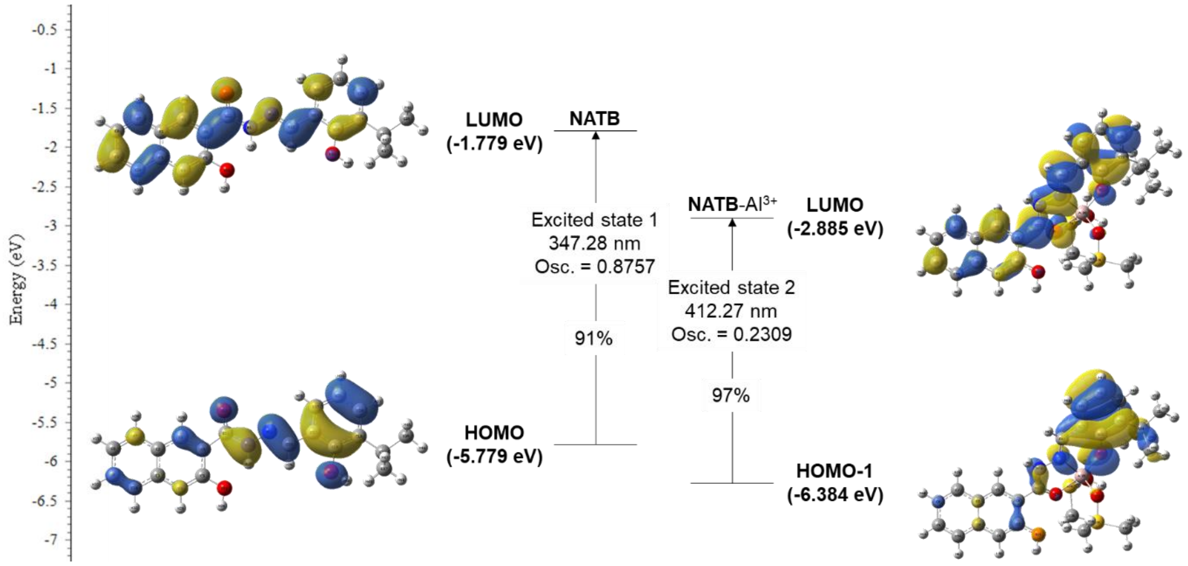

Figure S10. The major molecular orbital transitions and excitation energies of **NATB** and **NATB**-Al^{3+}.

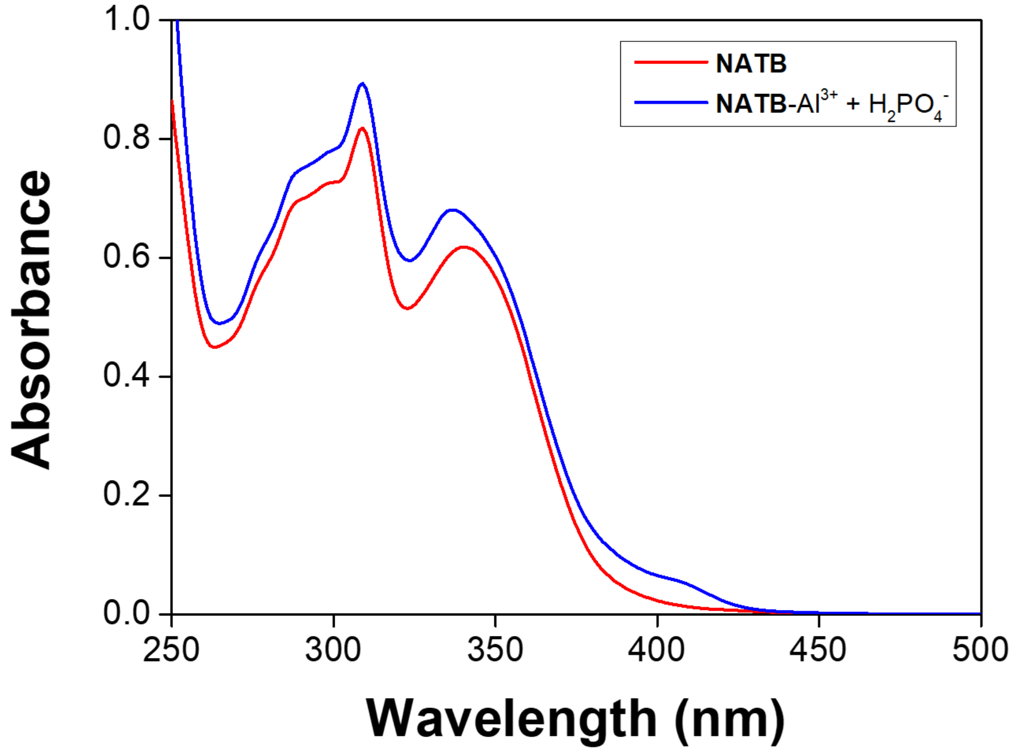

Figure S11. UV-vis spectra of **NATB** and **NATB**-Al^{3+} with $H_2PO_4^-$ in MeOH, respectively.

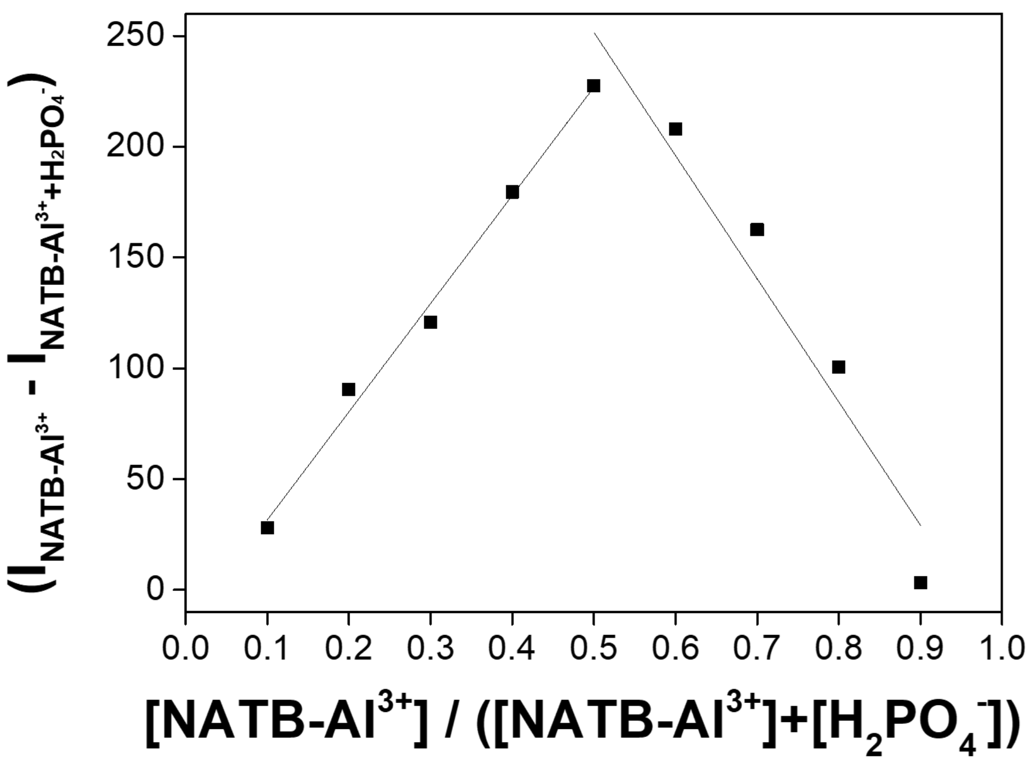

Figure S12. Job plot for the stoichiometry of **NATB**-Al^{3+} with $H_2PO_4^-$ (30 μM) in MeOH. Fluorescence intensity at 526 nm was plotted as a function of the molar ratio of [**NATB-**Al^{3+}] / ([**NATB-**Al^{3+}] + [$H_2PO_4^-$]).

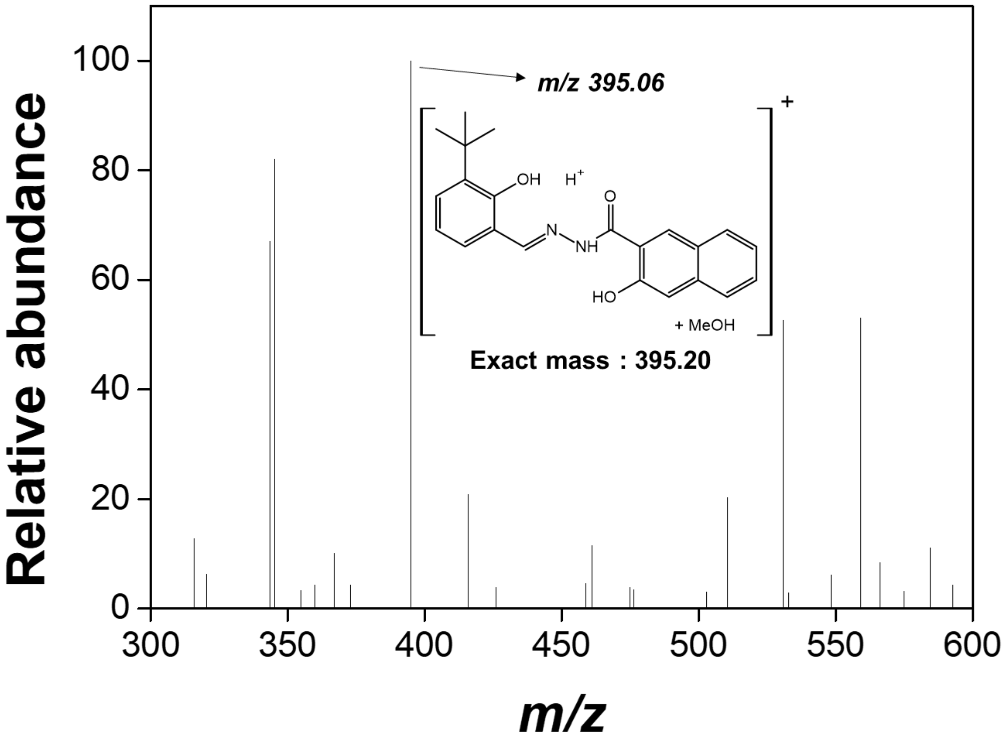

Figure S13. Positive-ion ESI-mass spectrum of **NATB-**Al^{3+} (100 μM) in MeOH upon the addition of 1 equiv of $H_2PO_4^-$ in H_2O.

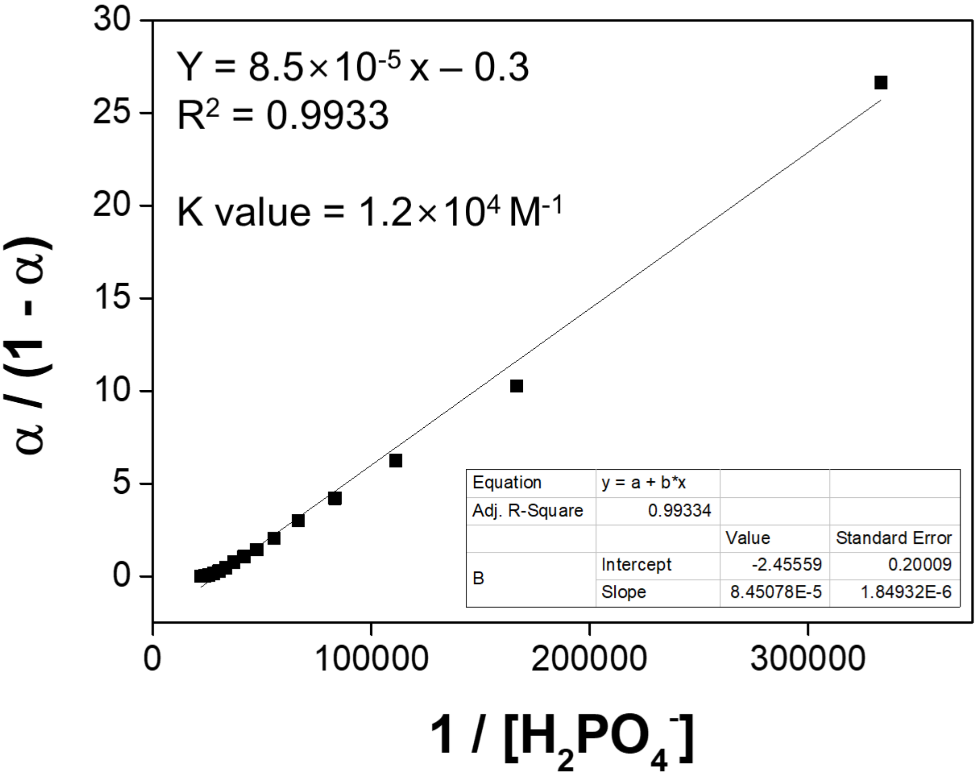

Figure S14. Li's equation plot (at 526 nm) of **NATB**-Al^{3+} (30 μM) based on fluorescence titration in MeOH, assuming 1:1 stoichiometry for the association between **NATB**-Al^{3+} and $H_2PO_4^-$.

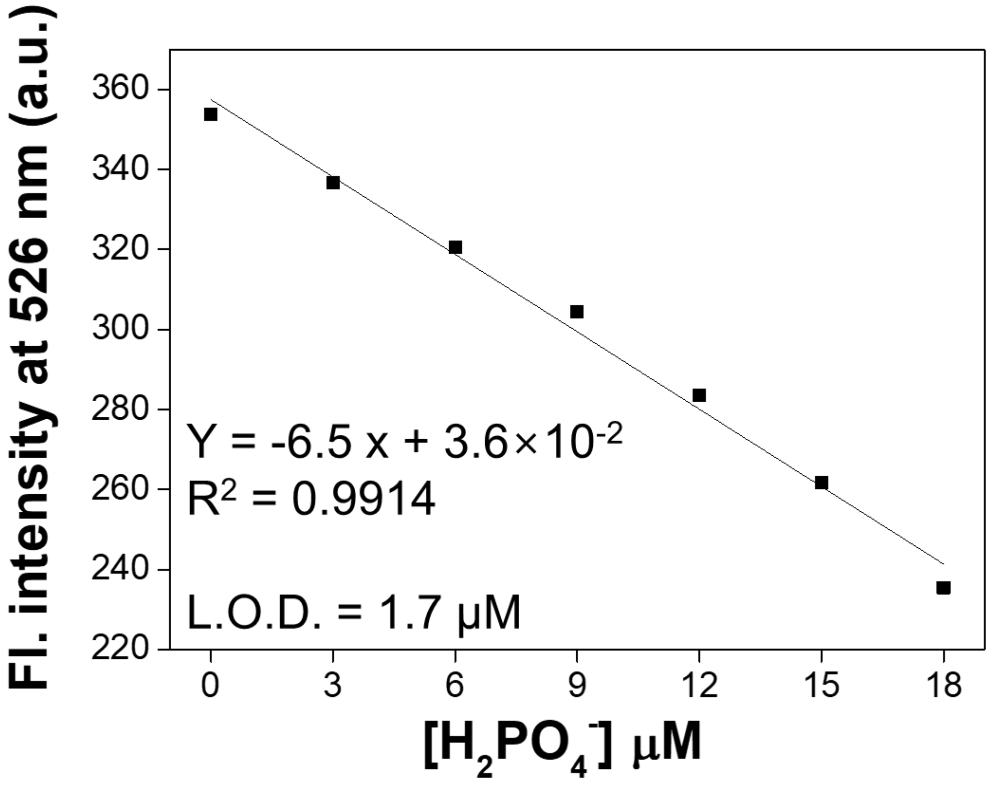

Figure S15. Calibration curve of **NATB**-Al^{3+} in MeOH as a function of $H_2PO_4^-$ concentration. [**NATB**-Al^{3+}] = 30 μM and [$H_2PO_4^-$] = 0.0 – 18.0 μM (λ_{ex} = 358 nm).

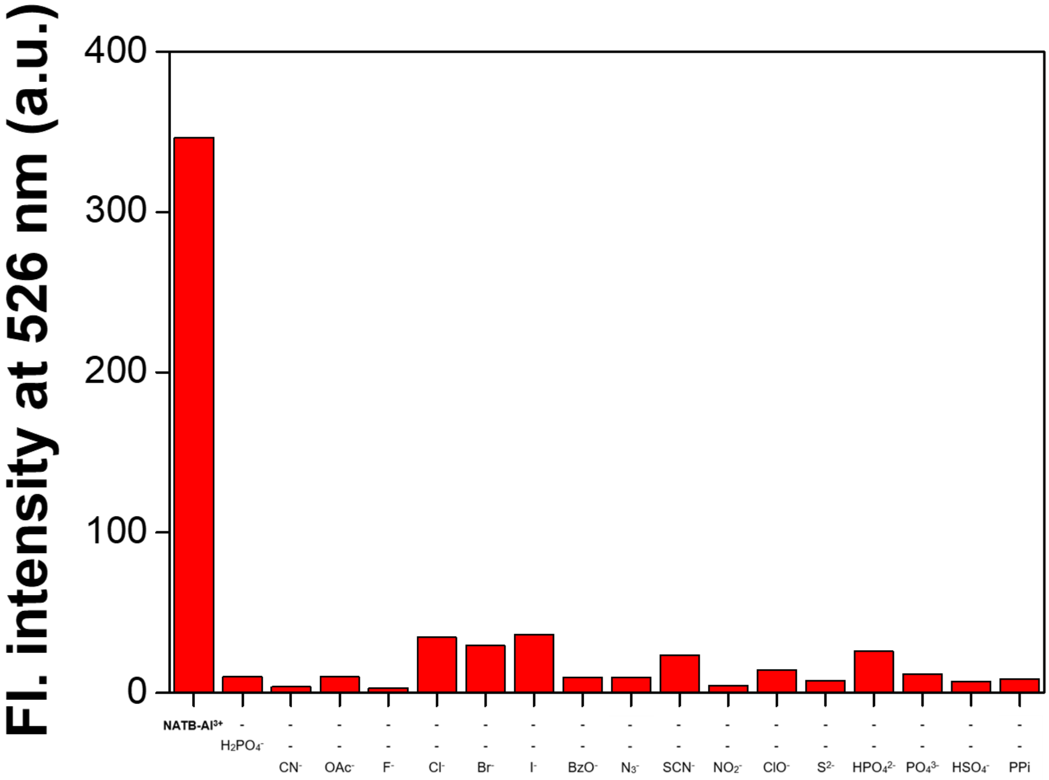

Figure S16. Interference studies of **NATB**-Al^{3+} (30 μM) toward $H_2PO_4^-$ (45 μM) in the presence of other anions (45 μM, λ_{ex} = 358 nm) in MeOH.
